# Exploring the Global Trends of *Bacillus*, *Trichoderma* and Entomopathogenic Fungi for Pathogen and Pest Control in Chili Cultivation

**DOI:** 10.1016/j.sjbs.2024.104046

**Published:** 2024-06-12

**Authors:** Muhamad Firdaus Syahmi Sam-on, Shuhaimi Mustafa, Mohd Termizi Yusof, Amalia Mohd Hashim, Ku Nur Azwa Ku Aizuddin

**Affiliations:** a Department of Food Sciences, Faculty of Science and Technology, Universiti Kebangsaan Malaysia, 43600 UKM Bangi, Selangor, Malaysia; b Department of Microbiology, Faculty of Biotechnology and Biomolecular Sciences, Universiti Putra Malaysia, 43400 Serdang, Selangor, Malaysia; c Department of Cell and Molecular Biology, Faculty of Biotechnology and Biomolecular Sciences, Universiti Putra Malaysia, 43400 Serdang, Selangor, Malaysia

**Keywords:** Biocontrol, Chili farming, Chemical pesticides, Microbes

## Abstract

Chili, renowned globally and deeply ingrained in various cultures. Regrettably, the onset of diseases instigated by pests and pathogens has inflicted substantial losses on chili crops, with some farms experiencing complete production decimation. Challenges confronting chili cultivation include threats from pathogenic microbes like *Xanthomonas, Fusarium, Phytophthora, Verticillium, Rhizoctonia, Colletotrichium* and Viruses, alongside pests such as whiteflies, mites, thrips, aphids, and fruit flies. While conventional farming practices often resort to chemical pesticides to combat these challenges, their utilization poses substantial risks to both human health and the environment. In response to this pressing issue, this review aims to evaluate the potential of microbe-based biological control as eco-friendly alternatives to chemical pesticides for chili cultivation. Biocontrol agents such as *Bacillus* spp., *Trichoderma* spp., and entomopathogenic fungi present safer and more environmentally sustainable alternatives to chemical pesticides. However, despite the recognised potential of biocontrol agents, research on their efficacy in controlling the array of pests and pathogens affecting chili farming remains limited. This review addresses this gap by evaluating the efficiency of biocontrol agents, drawing insights from existing studies conducted in other crop systems, regarding pest and pathogen management. Notably, an analysis of Scopus publications revealed fewer than 30 publications in 2023 focused on these three microbial agents. Intriguingly, India, as the world’s largest chili producer, leads in the number of publications concerning *Bacillus* spp., *Trichoderma* spp., and entomopathogenic fungi in chili cultivation. Further research on microbial agents is imperative to mitigate infections and reduce reliance on chemical pesticides for sustainable chili production.

## 1 Introduction

Chili, often referred to as hot pepper or chili pepper, stands among the world's most renowned crops. It belongs to the nightshade family and is commonly used as a spice, seasoning, or condiment in a wide variety of dishes (Liu et al., 2016, Yadav et al., 2021). Chili comes in diverse forms, varying in size, colour, and spiciness levels. Some of the most popular types include cayenne pepper, jalapeño, habanero, serrano, and Thai chili (Barbero et al., 2014; Chuaysrinule et al., 2020, Lozada et al., 2023). Additionally, chili offers various health benefits, serving as a good source of vitamins A and C, as well as antioxidants (Azlan et al., 2022). With the global population on the rise, the demand for chili has grown exponentially worldwide. Chili boasts a rich cultural history, often entwined with specific places and culinary traditions. It plays a significant role in numerous traditional recipes and festive occasions, making its presence felt worldwide.

**Fig. 1:**
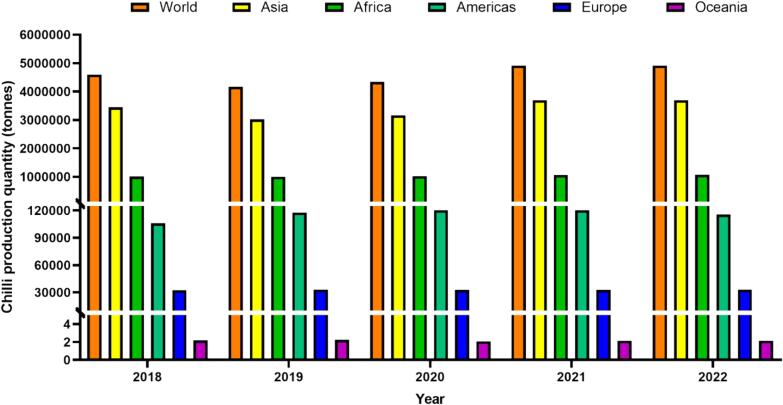
**Trends in Chili production quantity worldwide over the past 5 years (2018**–**2022).** The data, retrieved from the FAOSTAT website (https://www.fao.org /faostat/en/#data/QCL) on February 26th, 2024, was reported by the Food and Agriculture Organization of the United Nations, with 2022 being the latest available information. The dataset is categorized into six settings: World, Asia, Africa, Americas (combination of North and South America), Europe and Oceania. Four key sections were considered in the FAOSTAT query: year, countries, production quantity, and Chilies and peppers, dry (*Capsicum* spp., *Pimenta* spp.).

**Fig. 2:**
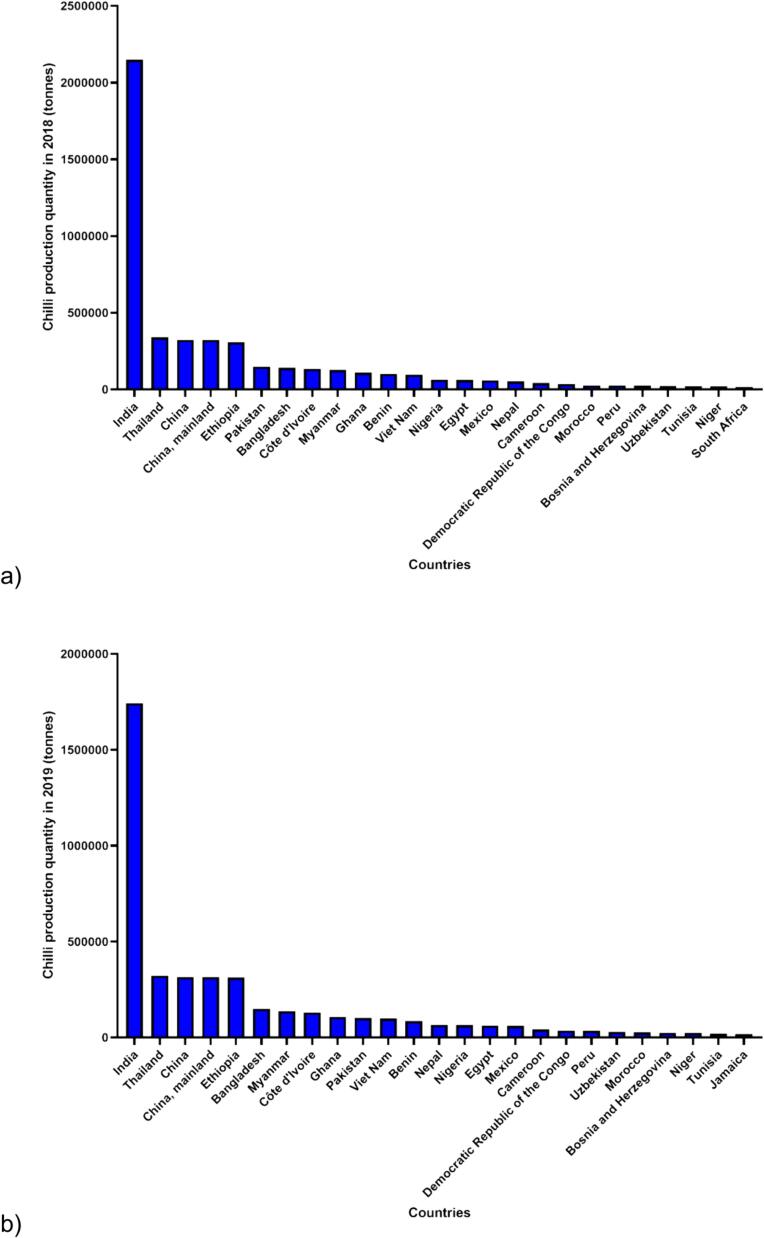

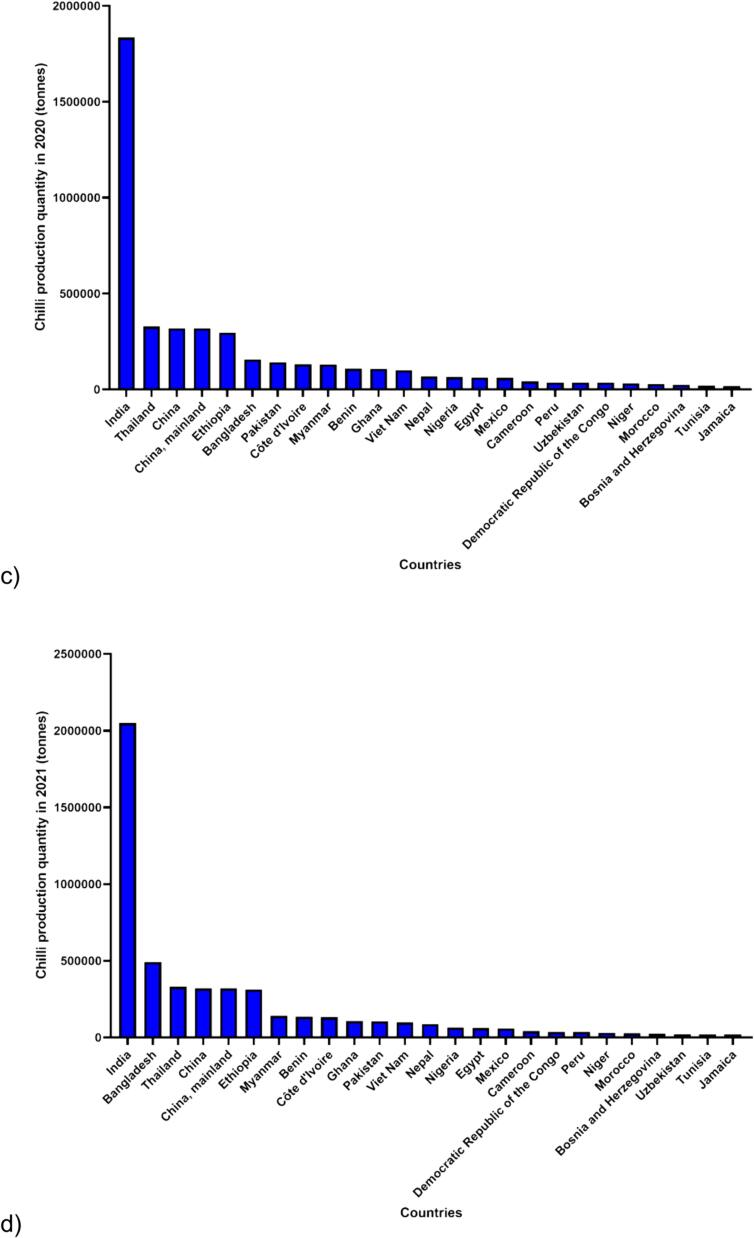

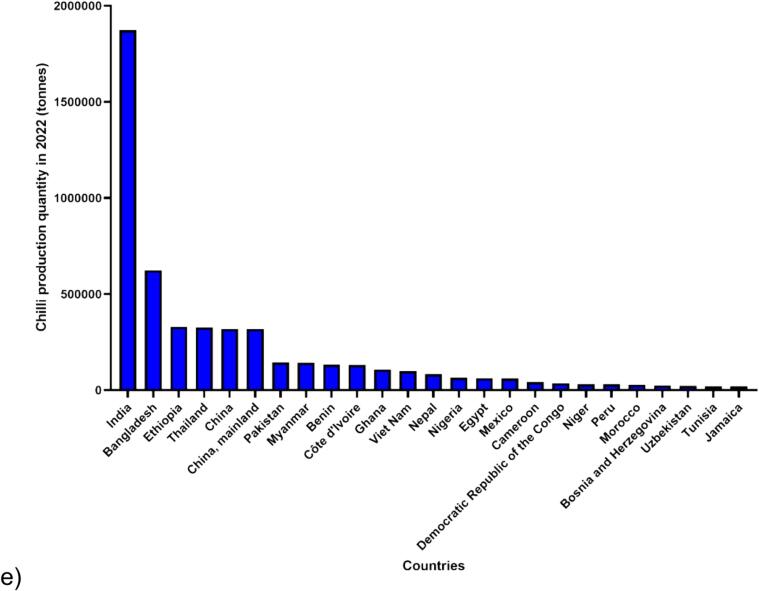
**Trends in Chili production quantity among the top 25 countries over the past 5 years (2018**–**2022).** The data, retrieved from the FAOSTAT website (https://www.fao.org/faostat/en/#data/QCL) on February 26th, 2024, was reported by the Food and Agriculture Organization of the United Nations, with 2022 being the latest available information. The dataset is categorized into five years: a) 2018, b) 2019, c) 2020, d) 2021, and e) 2022. Four key sections were considered in the FAOSTAT query: year, countries, production quantity, and Chilies and peppers, dry (*Capsicum* spp., *Pimenta* spp.).

FAOSTAT (2024) stated that the worldwide production of chilies demonstrated gradual increases over the five-year period, reaching a total production of 4.60, 4.17, 4.34, 4.91, 4.91 million tonnes in 2018, 2019, 2020, 2021 and 2022, respectively. In 2022 alone, Asia exhibited the highest chili production among continents, totalling 3.69 million tonnes. Following Asia, the production for Africa, the Americas, Europe, and Oceania were 1.07, 0.12, 0.03, and 0.000002 million tonnes, respectively (Fig. 1). Notably, Asia contributed to more than 70 % of the world's chili production in 5-year periods. Moreover, throughout this period, India consistently emerged as the leading producer, with production quantities of 2.15, 1.74, 1.84, 2.05 and 1.87 million tonnes in 2018, 2019, 2020, 2021 and 2022, respectively (Fig. 2). India not only dominates chili production but also stands out as the foremost exporter of dried chilies (Jalgaonkar et al., 2022, Kumar et al., 2022).

Even though the production of global Chilies has significantly increased in the past 5 years (2018–2022), the crops still impacted by pests and pathogens, including whiteflies (Hussain et al., 2022), thrips (Panyasiri et al., 2022), broad mites (Latha and Hunumanthraya, 2018), aphids (Xiao et al., 2020), fruit flies (Syamsudin et al., 2022), *Colletotrichum* (Mongkolporn and Taylor, 2018), *Phytophthora* (Mohammadbagheri et al., 2022), *Fusarium* (Bashir et al., 2018), *Xanthomonas* (Utami et al., 2022), and viruses (Abirami et al., 2022). To address these issues, chemical pesticides have been widely used, leading to the development of pesticide resistance among pests and pathogens (Hawkins et al., 2019). However, the concerning aspect lies in the potential hazards posed by chemical pesticides to farmers and the contamination they may inflict upon harvested chili crops (Khatun et al., 2023). In response to these challenges, extensive research has been directed towards the utilisation of biocontrol agents, particularly microbes, to overcome the limitations associated with chemical pesticides (Chin et al., 2022, Pawaskar and Kerkar, 2021, Yadav et al., 2021). Moreover, the available data on the study of microbial control agents in Chilies is also limited. Therefore, this review aims to evaluate the potential of microbe-based biological control as eco-friendly alternatives to chemical pesticides for chili cultivation.

## 2 Pathogens in chili plantations

**Fig. 3:**
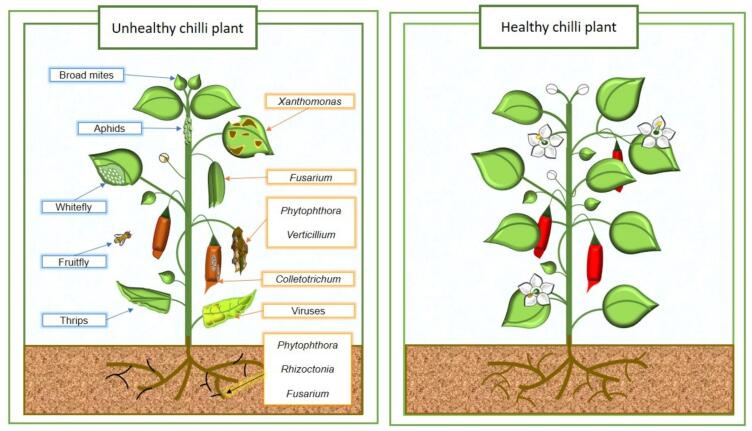
**Comparative illustration of unhealthy and health Chili plants.** The unhealthy Chili plant showed infection caused by pests and pathogens. The blue box indicates the pest in the unhealthy Chili plant: Broad mites, Aphids, Whitefly, Fruit fly and Thrips. Moreover, the orange box signifies the pathogens such as *Xanthomonas*, *Fusarium*, *Phytophthora*, *Verticillium*, *Rhizoctonia*, *Colletotrichium* and Viruses. (For interpretation of the references to color in this figure legend, the reader is referred to the web version of this article.)

Even though FAOSTAT (2024) data on worldwide chili production quantities showed an increase in the past five years (2018–2022), farmers worldwide still face challenges in chili plantations due to diseases that can harm production and result in significant economic losses (Oo and Oh, 2016, Saxena et al., 2016, Islam et al., 2020). It is important to identify the potential pathogens in chili plantations for disease prevention and treatment. Fig. 3 shows that common pathogens reported in chili crops including fungi (*Colletotrichum*, *Phytophthora, Fusarium, Sclerotium, Verticillium and Rhizoctonia*), bacteria (*Xanthomonas*) and viruses (Pepper Huasteco Yellow Vein Virus, Pepper Golden Mosaic Virus, Bell Pepper Endornavirus and Chili Leaf Curl Virus). This section will extensively discuss pathogens and their effects on chili plantations.

### 2.1 *Colletotrichum* spp

*Colletotrichum* spp. are pathogenic fungi responsible for inducing Chili anthracnose. The highest prevalence of this disease is typically observed in tropical or subtropical countries (Singh et al., 2022). Environmental factors, including heat and humidity, contribute to the disease's propagation (Banya et al., 2020). Three major Chili anthracnose species commonly reported in Southeast Asia which are *C. truncatum*, *C. siamense* and *C. scovillei* (Weir et al., 2012, Cannon et al., 2012). The pathogenicity of *Colletotrichum* spp. is generally evaluated on detached chili fruits, either pre- or post-wounding before inoculation. However, this approach overlooks the importance of the fruit's cuticle and epidermal cells, which serve as the first line of defense against pathogen invasion (Mongkolporn and Taylor, 2018).

*Colletotrichum* spp. can infect chili plants at any stage of development, with the fruit being particularly vulnerable (Saxena et al., 2016). Infected seeds result in pre- and post-emergence mortality of seedlings upon fungal germination. Although leaves can also be infected, pathogens enter a quiescent state in young leaves, remaining asymptomatic. Infected leaves remain healthy until they begin to senesce, at which point anthracnose symptoms become apparent. The infected senesced leaves serve as the primary source of inoculum for subsequent infections of both fruit and leaves (Ali et al., 2014, De Silva et al., 2017). Moreover, *Colletotrichum* species can develop micro-sclerotia to allow dormancy in soil during winter or harsh conditions, which can last for years. Conidia from acervuli and micro-sclerotia are transferred from disease to healthy fruit and foliage through rain or irrigation during warm and rainy periods (Banya et al., 2020). This infection-initiated Anthracnose symptoms manifest as sunken necrotic lesions with concentric rings of black acervuli on ripe chili fruit (Mongkolporn and Taylor, 2018).

### 2.2 *Phytophthora* spp

One of the most destructive diseases affecting crops worldwide, *Phytophthora* blight, is caused by the oomycete *Phytophthora capsici* (Mo, 2013; Li et al., 2020). This disease primarily targets tomato, bell pepper, and chili plants (Lamour et al., 2012, Mohammadbagheri et al., 2022). Infected chili plants exhibit several discernible symptoms, including root and crown rot, greyish-brown water-soaked lesions on leaves, as well as black stem and fruit lesions (Kamoun et al., 2015). Furthermore, disease severity is influenced by the maturity of the plant, with more mature plants generally displaying greater resistance compared to seedlings or early fruits (Mansfeld et al., 2017, Barchenger et al., 2018). *Phytophthora capsici* reproduces asexually by producing sporangia and motile zoospores when exposed to water, such as through rainfall or irrigation. Each sporangium can generate between 20 and 40 zoospores, which spread in standing water and infect neighbouring plants. Its rapid dissemination within a field can result in losses of up to 100 % within a matter of days (Barchenger et al., 2018; Moreira-Morrillo et al., 2022). The annual global losses attributed to this disease exceed USD 100 million (Barchenger et al., 2017).

### 2.3 *Fusarium* spp

*Fusarium* spp., including *Fusarium oxysporum f. sp. capsica*, is the fungal species responsible for causing Fusarium wilt in chili plantations (Jean et al., 2013, Bashir et al., 2018). This fungus is capable of long-term survival in the soil, even in the absence of its host plant. Infection occurs when the fungus infiltrates the plant through its roots, subsequently colonizing the plant's vascular system and impeding the transport of water and nutrients. The disease is more prevalent in warm and humid environments and can rapidly spread through contaminated soil, water, and plant debris (Muhammad et al., 2022). One significant morphological characteristic of *Fusarium* species aiding in their identification is the production of macro and microconidia, which are asexual spores of various shapes and sizes (Hami et al., 2021). The formation of additional structures known as chlamydospore spores ensures the pathogen's prolonged survival in both soil and plants, making disease management and prevention challenging (Raghu et al., 2016; El Kichaoui et al., 2017).

*Fusarium* wilt can have disastrous consequences for chili plants, leading to reduced growth, wilting, and leaf yellowing (Abdelaziz et al., 2022). Additionally, the leaves may become deformed, and the fruits may appear misshapen or small. In severe cases, the infected plant may succumb to the disease. *Fusarium* wilt significantly diminishes the output and quality of chili crops, causing farmers to incur substantial economic losses (Muhammad et al., 2022). Depending on the chosen cultivars and environmental factors, the overall yield loss caused by *Fusarium* wilt has been estimated to range from 10 to 80 % in global chili production (Singh et al., 2017, Girma, 2022). This disease poses significant threats to chili production by reducing growth, fruit yield, and quality (Iftikhar et al., 2022).

### 2.4 *Sclerotium* spp

Sclerotium rot, caused by *Sclerotium rolfsii* Sacc., is one of the most devastating soil-borne diseases, posing a significant challenge to the successful cultivation of chili crops (Mao et al., 1998, Mandal et al., 2023). *S. rolfsii* is frequently found in tropical and subtropical regions with high temperatures during the rainy season. This fungus can infect chili plants from the nursery stage to maturity, affecting leaves, fruit, twigs, stems, and even seeds. Southern blight, commonly known as Sclerotium rot or stem rot, has a wide geographic range in warm regions. Capsicum plants are particularly vulnerable to this disease, with reported fatality rates of 50–60 % (Naresh et al., 2022). Plants infected with this pathogen will rot, wilt, and inevitably die. Controlling these diseases is difficult because the pathogens infect plants from the soil, have a wide host range, and can persist in the soil for long periods (Murthy et al., 2018). As a result, chemical control methods have been inefficient in managing these infections.

### 2.5 *Verticillium* spp

Vascular wilt diseases caused by *Verticillium dahliae* Kleb. is an economically significant soil disease that reduces yields in a variety of crop species, including chili. This pathogen, *V. dahliae* can infect plants at any stage of development, primarily targeting organs and tissues that absorb and transport water and nutrients (Kowalska, 2021). Controlling the spread of Verticillium wilt in plants is difficult due to the persistent dormant structure of microsclerotia, long-term variability, and coevolutionary relationships with host plants (Ju et al., 2022). *V. dahliae* infects chili plants at any stage of development. Yellowing and drooping leaves are common symptoms, which might occur on a few branches or across the plant. Infected plants show inward rolling of the leaf margins, followed by foliar wilting. Severely diseased plants exhibit brown foliage.

Chili plants infected with virulent strains of *V. dahliae* in greenhouse conditions or infected early in the season in the field suffer significant stunting and have small, yellow-green leaves. Consequently, withered leaves and shriveled fruits remain on plants that eventually perish. Brown staining of vascular tissue is observed when longitudinally cutting the roots and lower stem of wilted plants (Vasileva et al., 2019). Current methods, such as pesticide control, genetic breeding, and cropping pattern optimization, are limited. Disease resistance breeding is restricted by a long breeding cycle and a lack of natural resistance resources. Chemical management approaches are inefficient for soil-borne diseases including Verticillium wilt (Díaz-Rueda et al., 2021).

### 2.6 *Rhizoctonia* spp

*Rhizoctonia solani* Khan, the causal agent of root rot, is a major soil-borne pathogen of chili. It causes damping-off disease in seedlings as well as root and stem rot in young chili transplants. Root rot of chili is one of the most severe yield-destabilizing factors, leading to significant yield losses each year (Sid Ahmed et al., 2003, Varma and Kumhar, 2022). This disease also causes seed decay, damping-off, root and stem rot, canker, sheath blight, and rot in both monocot and dicot plants. *R. solani* has a remarkable ability to remain in soil saprophytically, with a diverse host range and little divergence between its specialized strains. This makes it particularly difficult to establish crop resistance in chili. Moreover, it can induce disease incidence of up to 33.2 % in seedlings grown in greenhouses and 40.2 % in the chili field (Abhinav et al., 2023). The root rot complex in chili is a significant pest. Yellowing and wilting in growing crops usually starts by light to dark brown lesions on the stem around the center. This is followed by drooping and wilting of the affected leaves, which eventually leads to wilting of the entire plant. Mature plants are prone to sudden dryness, while the infestation kills seedlings soon after the germination (Varma et al., 2020).

### 2.7 *Xanthomonas* spp

Bacterial Leaf Spot, caused by pathogenic *Xanthomonas* spp., stands out as one of the most economically devastating diseases affecting chili plants (Roach et al., 2018, Utami et al., 2022). *Xanthomonas* spp. can induce necrotic lesions and defoliation by infecting leaves, fruits, and stems. Xanthomonas infections give rise to leaf lesions surrounded by a yellow halo of varying sizes. This pathogenic bacterium infects plants in two different phases. The first phase is the epiphytic phase, during which bacterial cells are introduced to aerial surfaces such as leaves and fruits (An et al., 2020). Upon initial contact, bacteria enter through natural openings like stomata, wounds, or hydathodes (Hugouvieux et al., 1998). The endophytic phase occurs as bacteria multiply within the host tissue, leading to a significant increase in the bacterial cell population. The bacteria then re-emerge on the leaf's surface, where they are dispersed to new hosts by rain and wind, initiating a new cycle of infection. As the bacterial population reaches a critical mass, they infiltrate the mesophyll tissues, giving rise to disease symptoms on the leaves (Utami et al., 2022). Although these diseases do not directly reduce fruit quantity, the economic loss primarily stems from the diminished market value of the damaged fruit (Pajčin et al., 2020).

### 2.8 Pepper huasteco yellow vein virus and pepper golden mosaic virus

Pepper Huasteco Yellow Vein Virus (PHYVV) and Pepper Golden Mosaic Virus (PepGMV) are members of the genus Begomovirus, which are among the few DNA viruses from the family Geminiviridae that are frequently reported in America (Jo et al., 2017). It is widely distributed across Mexico and specifically targets chili crops (*Capsicum annuum* L.) (Rentería-Canett et al., 2011, Rivero-Montejo et al., 2023). Throughout Central America, Mexico, the Caribbean Basin, the Southern United States of America, and South America, begomoviruses have been documented as particularly devastating diseases to cultivated chili plants. Yield losses in these regions can range from 50 to 90 % of total production (Gracia-Medrano et al., 2022). Both PHYVV and PepGMV is transmitted by whitefly (*Bemisia tabaci* G), a vector that is widely distributed worldwide and is responsible for transmitting some of the most devastating Geminivirus diseases (Retes-Manjarrez et al., 2019). The primary symptoms of Begomovirus in chili plants include yellowing of veins, distortion of leaves, yellow mosaic patterns, leaf curling, stunted plant growth, and decreased yields (Retes-Manjarrez et al., 2018). Currently, chemical insecticides are the common approach to reduce the infections, as they control the population of whiteflies in chili crops.

### 2.9 Bell pepper endornavirus

The Bell pepper endornavirus (BPEV) falls within the Alphaendornavirus genus. It possesses a single linear single-stranded RNA genome of about 14 kb in size, consisting of a single open reading frame (Valverde et al., 2019). Endornaviruses, like hypoviruses (Hypoviridae), have a linear dsRNA genome with a single open reading frame and obvious helicase and polymerase domains. However, no real virions have been found in infected tissue. BPEV shows a high number of vertical transmission (up to 100 %) through the ovule compared to pollen. Consequently, when both parents are infected, the transmission rate is notably high (Okada et al., 2011, Safari and Roossinck, 2018). However, infections caused by these viruses often remain asymptomatic, posing significant challenges for detection. Presently, whole genome sequencing stands as the most effective method for detection, yet this technology remains inaccessible to many farmers. Further research into BPEV is imperative to identify the vectors responsible for transmitting the virus to hosts, particularly in chili crops.

### 2.10 Chili leaf curl virus

The primary cause of leaf curl disease in chili plants is the Chili Leaf Curl Virus (ChiLCV), classified under the Family Geminiviridae and Genus Begomovirus (Shingote et al., 2022). Moreover, it is important to know that global sustainability of chili production has been threatened by the resurgence of new Begomoviral strains arising from recombination and mutations, including ChiLCV (Juárez et al., 2019). ChiLCV is known to cause major problem of chili (*Capsicum* spp.) in Asian region which lead to 100 % losses in the crops (Kumar et al., 2011; Senanayake et al., 2012; Thakur et al., 2018). When combined with a thrips or mite infestation, chili leaf curl disease becomes even more severe (Menike and De Costa, 2017; Thakur et al., 2018). Khan et al., 2006 presented the initial evidence of the occurrence of the Tomato Leaf Curl New Delhi Virus in India. Recently, it was also observed that chili plants in India were naturally affected by mixed infections with the Chili Leaf Curl Virus, Cotton leaf curl Multan Virus, and Tomato Leaf Curl Gujrat Virus (Mishra et al., 2020). This pathogen has been extensively studied in chili crops, revealing its significant impact on global chili production. Current control methods largely revolve around insecticides that target the vectors of the chili leaf curl virus. However, due to the substantial losses experienced by chili farmers, excessive use of chemical pesticides has become commonplace, adversely affecting farmers, consumers, and the environment.

## 3 Pests in chili plantations

Besides pathogens, pests often pose a significant threat to chili crops by causing diseases. Moreover, many of these pests act as vector for different types of viruses, worsening the conditions of the diseases they spread. As shown in Fig. 3, some of the most frequently reported pests in chili plantations include whitefly, thrips, broad mite, aphids, and fruit fly. This section will focus on how these pests affect chili plants and contribute to the spread of toxins and viruses.

### 3.1 Whitefly

Whitefly (*Bemisia tabaci*) is a severe pest in chili plantations, directly feeding on the plants and can transmitting the viruses, resulting in production losses (Hussain et al., 2022). The whitefly attaches itself to the plant with a pedicel inserted into a tiny slit formed in the female's tissues and lays white eggs, usually in circular groups, on the underside of leaves. The first instar, also referred to as the “crawler,” is the only mobile nymphal stage upon hatching. It appears flat, round, and scale-like. Once the crawlers find a suitable feeding spot on the underside of the leaf, they become sessile, feeding there, and lose their legs during the subsequent moult (Jeevanandham et al., 2018). Additionally, whitefly can transmit viruses that are harmful to plants, including the tomato yellow leaf curl virus, which can significantly impact chili crop production (Ghosh et al., 2022). When a whitefly feeds on an infected plant and then moves to a healthy one, it spreads the virus, thus, Marchant et al., 2022, Guo et al., 2019, and Ghosh et al. (2019) have all confirmed that whiteflies carry the tomato yellow leaf curl virus. Infected plants show stunted growth, reduced fruit production, and may appear misshapen or discoloured. Furthermore, whiteflies have a rapid reproductive rate, leading to population explosions in a short period of time (Prasad et al., 2020).

### 3.2 Thrips

Chili thrips (*Scirtothrips dorsalis*) are significant pests that impact over 100 plant species globally, including various vegetable crops such as cucumber, pepper, and eggplant (Panyasiri et al., 2022). These insects consume all parts of the plant, with a particular preference for young leaves, buds, and fruits, leading to necrosis by depleting the contents of epidermal cells (Nagaraju and Kumar, 2022). Feeding by thrips on leaves results in changes in tissue color from silver to brown and black. Furthermore, this insect can transmit seven viral diseases, including the Chili leaf curl virus, peanut necrosis virus, and tobacco streak virus in groundnut crops (Panyasiri et al., 2022). Identifying thrips in new vegetation poses a challenge due to their small size (2 mm) and rapid mobility. The minute eggs can survive for up to a week within delicate plant tissues. Typical first and second-instar larvae measure between 0.36–0.38 mm and 0.68–0.71 mm, respectively, while pupae can range from 0.78 to 0.80 mm in size (Deka et al., 2020, Kaur and Lahiri, 2022). Adult thrips have pale yellow bodies with dark brown antecostal ridges on their tergites and sternites. An average adult thrips measures less than 1.5 mm in size and features wings with black fringes (Deka et al., 2020).

### 3.3 Broad mite

The broad mite (*Polyphagotarsonemus latus*) is a significant pest that severely impacts chili production, leading to reduced yield and economic losses. This pest deposits its eggs on the undersides of leaves and affects actively growing plant tissues, including leaves, flowers, shoots, and other plant parts. Moreover, due to their small and wide size (0.1–0.2 mm long), mites can often go unnoticed at the initial stages of an outbreak. It is not until the affected plants display damage that mite infestation is noticed (Rodríguez et al., 2017, Cabedo-López et al., 2021). While feeding, the mites inject toxins that result in stunted plants with short internodes (Pal and Karmakar, 2017). Plants afflicted by broad mite infestations exhibit coppery and dark leaves, along with leaf curling. More critically, these plants produce smaller, inedible fruits (Patavardhan et al., 2020). Mite-induced damage can manifest in various symptoms, with some researchers attributing these symptoms to the toxicity of the mite's saliva and the host plant's defense mechanisms. However, these symptoms may be mistaken for viral infections, herbicide damage, nutrient deficiencies, or physiological issues. Variability in these symptoms could be linked to species complexes or feeding ecotypes (Ovando-Garay et al., 2022). Mite infestation diminishes the quality of chili fruits and seeds, leading to a production loss of over 60 % (Nasrin et al., 2020). Consequently, the yield loss in chili cultivation due to mite infestation can be as high as 96.4 % (Latha and Hunumanthraya, 2018).

### 3.4 Aphids

Aphids, also known as greenflies, blackflies, plant lice, ant cows, and various other names, represent a general term for all insects in the Aphididae family (order Hemiptera, suborder Sternorrhyncha, infraorder Aphidomorpha, superfamily Aphidoidea) which more than 4000 species have been identified (Singh and Singh, 2021, Sharawi, 2023). These small (1–10 mm) soft-bodied sap-sucking insects locate their hosts through olfactory, visual, tactile, and gustatory cues. Plant surface components such as the waxy cuticle and trichomes release deterrents and toxic metabolites, while the apoplastic space serves as another location where the stylet may encounter plant defenses (Nalam et al., 2019). Aphid adults and nymphs extract sap from pepper plants, leading to leaf curling and yellowing. Additionally, they secrete honeydew onto the leaves of pepper plants, causing sooty Mold and interfering with the plant photosynthesis (Xiao et al., 2020).

Furthermore, when the quality of their host deteriorates, aphids develop winged forms to facilitate dispersal to new plants. These characteristics, coupled with their capacity to infest nearly any part of the plant, consume large quantities of photo assimilates, disrupt source-sink patterns, transmit over 200 viral diseases, and develop resistance to a wide range of insecticides, make them extraordinary pests (Harris and Maramorosch, 2014, Bass and Nauen, 2023). These insects thrive within specific temperature ranges, which can vary across and within species (Ge et al., 2023). The selection, settlement, and establishment of feeding on a new host plant are crucial for an aphid's fitness and the evolution of plant resistance (Züst and Agrawal, 2016, Shih et al., 2023).

### 3.5 Fruit fly

*Bactrocera dorsalis*, an Oriental fruit fly, is another pest that impedes the growth of chili plants (Syamsudin et al., 2022). During the developmental stage, its eggs exhibit resistance to both high and low temperatures. Female insects lay eggs by inserting them into the fruit skin, leaving discernible black dots from the ovipositor markings on infested pepper fruits. After 2–3 days, the eggs hatch, and the newly hatched larvae feed and mature within the fruits. As the third larval instar approaches, the nymphs exit the fruits, descending to the ground to pupate in the soil (Said et al., 2017). By this stage, the fruits are already damaged, prematurely detached from the plants, and rendered unmarketable. The pupal stage lasts approximately ten days, after which the adults emerge from the ground, seeking protein-rich foods before mating (Drew and Romig, 2013). According to several studies, the rate of chili fruit loss due to fruit flies ranges between 40 and 60 %, with the possibility of reaching 100 % loss if fruit fly control measures are not implemented (Hasyim et al., 2014, Said et al., 2017, Rusaldy, 2023). Although there are no reports of fruit flies acting as vectors for viral transmission in chili crops, studies have shown that fruit flies can carry viruses from several genera and families, including Dicistroviridae, negev-like virus clades, Thika virus clades, Solemoviridae, Narnaviridae, Nodaviridae, Iflaviridae, Orthomyxoviridae, Bunyavirales, Partitiviridae, and Reoviridae (Zhang et al., 2022). Therefore, controlling fruit flies is essential to prevent the potential emergence of novel viruses that could infect chili plants.

## 4 Chili disease management using biocontrol agent

Biological control is “a form of pest control that uses living organisms to suppress pest densities to lower levels. It is a form of ecologically based pest management that uses one kind of organism (the ”natural enemies“) to control another (the pest species)” (Hoddle and Van Driesche, 2009). These approaches are an effective and sustainable alternative to chemical pesticides, which can harm the environment and human health (Hough et al., 2022). This section will highlight three biocontrol agents for chemical treatment alternatives using microbes, including *Bacillus* spp., *Trichoderma* spp. and entomopathogenic fungi. Despite some challenges, using biocontrol in Chili farming offers a sustainable and effective means of pest and pathogen control that can improve crop yield and environmental health. Although this review will focus on *Bacillus*, *Trichoderma*, and entomopathogenic fungi, other biocontrol agents have also been studied against chili pathogens. These include plant growth-promoting rhizobacteria, calcium (Maxifos Ca), *Ascophyllum nodosum* (Greencal), *Arthrospira platensis*, endophytic fungi *Aspergillus*, cyanobacteria-mediated immune responses, and Ziziphus spina-christi leaves' extract containing silver nanoparticles (Abdelaziz et al., 2022; Attia et al., 2022, Abdelaziz et al., 2023a, Attia et al., 2023, Abdelaziz et al., 2023b).

### 4.1 *Bacillus* spp

*Bacillus* spp. is highly used as a biocontrol agent for crops, including Chilies (Dowarah et al., 2021, Yanti et al., 2020). A comprehensive overview of various strains of *Bacillus* and their efficacy against pathogenic microbes in Chili farming was presented in Table 1. For instance, Zou et al. (2024) demonstrated that *Bacillus velezensis* LY7 induces hormone synthesis in chili plants, thereby enhancing resistance against *Colletotrichum scovillei* while promoting plant growth. Kumar et al. (2021) elucidated the effectiveness of *Bacillus subtilis* AKP in suppressing *Colletotrichum capsici* infections in chili plants, suggesting its potential as a growth-promoting agent. Montoya-Martínez et al. (2023) identified *Bacillus cabrialesii* subsp. tritici TSO2T as a promising biocontrol agent against *Fusarium languescens*, the causative agent of Fusarium wilt in chilies. Qiao et al. (2023) reported significant biocontrol activity of *Bacillus subtilis* PTS-394 against *Fusarium solani*.

**Table 1:** *Bacillus* against pests and pathogens in Chilies farming.

***Bacillus***	**Pest/Pathogen**	**Remarks**	**Reference**
*Bacillus velezensis* LY7	*Colletotrichum scovillei*	*B. velezensis* LY7 induces the hormone synthesis in Chilies plants which enhanced disease resistance against *C. scovillei* and promotes growth.	Zou et al. (2024)
*Bacillus subtilis AKP*	*Colletotrichum capsici*	*B. subtilis* AKP can suppress the *C. capsica* infection in Chilies, through *in vitro*, molecular, and *in vivo* analyses, in addition to act as potential plant growth promoting agent.	Kumar et al. (2021)
*Bacillus cabrialesii* subsp. tritici TSO2T	*Fusarium languescens* CE2	*B. cabrialesii* subsp. tritici TSO2T shown potential biocontrol agent against *F. languescens*, a causative agent of *Fusarium* wilt in Chilies, through phenotypic and metabolomic analyses.	Montoya-Martínez et al. (2023)
*Bacillus subtilis* PTS-394	*Fusarium solani*	*B. subtilis* PTS-394 shown a good biocontrol activity against *Fusarium solani* at 63 % and 74.43 %.	Qiao et al. (2023)
*Bacillus cereus* F-BC26 and *Bacillus thuringiensis* F-BT24	*Xanthomonas euvesicatoria*	F-BT24 and F-BC26 strains formulations promoted Chilies growth and protected it against *Xanthomonas euvesicatoria*, a causative agent of bacterial spot disease.	Hernández-Huerta et al. (2023)
*Bacillus* spp. isolates (SK, CM) and *Bacillus thuringiensis*	*Xanthomonas vesicatoria*	*Bacillus* spp. strain SK and CM, with *B. thuringiensis* has the potential antagonistic activity against *X. vesicatoria* in both local and distant tissues.	Aziz et al., 2024
*Bacillus licheniformis* BL06	*Phytophthora capsici*	*B. lincheniformis* BL06 reduced motility and demonstrated lytic activity against *P. capsici* zoospores in chilies tissues.	Li et al. (2020)
*Bacillus subtilis* AH18 and *Bacillus licheniformis K*11	*Phytophthora capsici*	Both *Bacillus* microbial agents *in vivo* synergistically suppressed Phytophthora blight at 80 % in chilies farming.	Lim and Kim (2010)
*Bacillus subtilis* FZB27	Cucumber mosaic virus	*B. subtilis* FZB27 can improved the growth and productivity of pepper plants infected with cucumber mosaic virus	Moalla et al. (2020)
*Bacillus amyloliquefaciens* 5B6	Cucumber mosaic virus	Strain 5B6 shown high biocontrol activity against viruses by boosting defense priming of salicylic acid and jasmonic acid signalling in chilies under field settings.	Lee and Ryu (2016)

Furthermore, Hernández-Huerta et al. (2023) demonstrated that formulations of *Bacillus cereus* F-BC26 and *Bacillus thuringiensis* F-BT24 strains stimulate chili growth and protect against *Xanthomonas euvesicatoria*, the causative agent of bacterial spot disease. Aziz et al. (2024) uncovered the potential antagonistic activity of *Bacillus* spp. strains SK and CM, in conjunction with *Bacillus thuringiensis*, against *Xanthomonas vesicatoria* in chili tissues. Li et al. (2020) revealed that *Bacillus licheniformis* BL06 reduces motility and exhibits lytic activity against *Phytophthora capsici* zoospores in chili tissues. Lim and Kim (2010) demonstrated the synergistic suppression of phytophthora blight by *Bacillus subtilis* AH18 and *Bacillus licheniformis K*11 in chili farming. Moreover, Moalla et al. (2020) illustrated that *Bacillus subtilis* FZB27 enhances the growth and productivity of pepper plants infected with cucumber mosaic virus, while Lee and Ryu (2016) underscored the high biocontrol activity of *Bacillus amyloliquefaciens* 5B6 against these viruses in chili plants.

Although there are no reports of *Bacillus* spp. effectively controlling pests such as whitefly, thrips, broad mites, aphids, and fruit flies in chili plants, other studies have demonstrated the efficacy of these Gram-positive bacteria in managing insects in different crop fields. Gómez-Gutiérrez et al. (2023) reported that *Bacillus* strain ROSS2 produced three biosurfactants with nematicidal and acaricidal activity, with 39.29 % mortality in nematodes (*Nacobbus aberrans*) and 57.97 % mortality in mites (*Tyrophagus putrescentiae*). Biosurfactants released by this *Bacillus* sp. can be used to biocontrol root-knot nematodes and mites. Álvarez-Lagazzi et al. (2021) stated that applying the *Bacillus subtilis* spore on the artificial diet and leaves significantly reduce the number of aphids in the grain crops. Moreover, Cuatlayotl-Cottier et al. (2022) reported that *Bacillus thuringiensis* spores had been utilized as biological insecticides against the aphids *Melanaphis sacchari*, *Rhophalosiphum maidis*, *Aphis fabae*, and *Tetranychus urticae* by 80 % and 90 % through *in vitro* and greenhouse approaches.

Additionally, a study by El-Gaied et al. (2020) discovered the first Vip3 gene from *Bacillus thuringiensis* that can be used to control whiteflies. The vip3 gene from an Egyptian strain of *B. thuringiensis* was tested against whitefly and shown to be very toxic, suggesting that it could be a potential biocontrol agent against whiteflies. Singh et al. (2021) confirmed that *Bacillus thuringiensis* produces insecticidal 'Cry' proteins that are poisonous to insect larvae of the orders Lepidoptera, Coleoptera, and Diptera, among others. The insecticidal effects of pure Cry protein on insect larvae of the lepidopteran and dipteran orders Tobacco cutworm (*Spodoptera litura*), Greater wax moth (*Galleria malonella*), fruit fly (*Bactrocera cucurbitae*), and Common house mosquito (*Culex pipens*) were significant. When larvae of all insects were exposed to Cry protein isolated from *B. thuringiensis* VIID1, there was a substantial increase in larval mortality, rising from 5.6 % in the control group to 78.4 % at a 10 μg/ml concentration.

Once spores and crystals of *B. thurengiensis* are consumed, Cry toxins can be released and activated from the crystal form and then converted by gut proteases. These toxins bind to specific receptors on the surface of intestinal cells, causing cell death and the loss of the gut epithelium. This damage allows the spores to germinate, and the vegetative bacteria can then enter the body cavity via the damaged gut lining, resulting in sepsis, which kills the targeted pest within 2 to 3 days (Nawrot-Esposito et al., 2020). The application of *Bacillus thuringiensis* on plantations for pest control is predominantly carried out using spray methods. This results in the presence of the bacteria or the Cry protein on the surfaces of the plants, including the stems, leaves, and fruit. However, for larvae of fruit flies that develop within the fruit, the mechanism by which *B. thuringiensis* or the Cry protein penetrates the fruit to inhibit the larvae remains unknown. Although studies have demonstrated that B*. thuringiensis* can effectively control fruit fly populations and have noted the presence of larvae within the fruit, there is a lack of detailed explanation regarding this process (Vidal-Quist et al., 2010, Buentello-Wong et al., 2015, Shishir et al., 2015; Singh et al., 2021). This gap in understanding necessitates further research to enhance the efficacy of *B. thuringiensis* in controlling fruit fly larvae and other pests in plantations, particularly in chili crops.

Even though the effectiveness of *Bacillus* spp. against pests and pathogens has been demonstrated in laboratory and field experiments, it is critical to determine the safety requirements of any isolate or product. According to Sam-on et al. (2022), antibiotic susceptibility and haemolysis tests are methods for proving the safety of bacteria. This safety analysis is critical because some bacteria, such as *Bacillus pumilus*, can either be harmless or pathogens to humans and crops (Wang et al., 2022, Habib and Ahmed, 2023). Furthermore, the researchers must appropriately characterise the bacteria to prevent harmful *Bacillus* spp., such as *Bacillus anthracis* (Hsieh and Stewart, 2023). Apart from sanger sequencing, which targets specific genes such as 16S rRNA, whole genome sequencing is a proper strategy to prove bacterial identity (Sam-on et al., 2023). Hence, there is a critical need for extensive research into the potential biocontrol activity of *Bacillus* spp. against pests and pathogens in chili farming. Such studies are essential to substantially diminish reliance on chemical pesticides and enhance the sustainability of ecosystems.

**Fig. 4:**
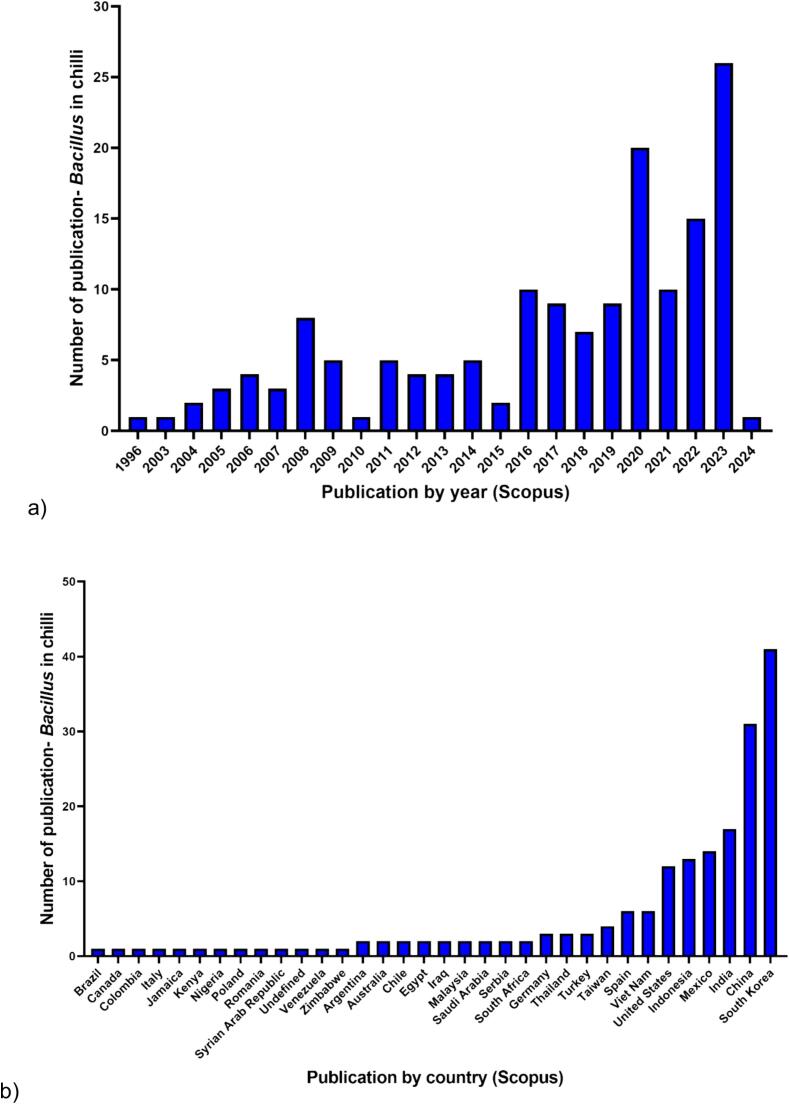
Data analytics on the application of *Bacillus* spp. in chili production. These data were collected based on the results from the Scopus website (https://www.scopus.com) on February 26th, 2024. The search query “*Bacillus* AND (Chili OR chili OR pepper)” was used to conduct an “article title” search on the Scopus website. The analysis focused on determining the number of publications by a) year and b) country.

Additionally, Scopus-based data analytics of *Bacillus* spp. related to chili production was showed in Fig. 4. According to the Scopus database, publications on *Bacillus* species have been documented since 1996. Currently, there are 150 published documents available on the Scopus website related to the application of *Bacillus* in chili production. Although the number of publications concerning *Bacillus* in chili production has steadily increased over the years, the total count remains relatively low, with the highest number not surpassing 25 documents in 2023. Moreover, the data analytics have been categorized by countries. The Scopus database has identified 33 countries and 1 undefined country in connection with *Bacillus* research in chili production. South Korea has demonstrated the highest number of publications, with 40 documents, followed by China and India with 30 and 16 documents, respectively. Malaysia has only contributed 2 publications on this topic, published by Gan et al. (2015) and Leong et al. (2021).

### 4.2 *Trichoderma* spp

United Stated Environmental Protection Agency (2007) stated that *Trichoderma* spp. is considered as safe and non-toxic to humans and plants as pesticides/fungicides. *Trichoderma* stands out as one of the most effective biological agents in fostering plant resistance. Through a sophisticated network of mechanisms, various *Trichoderma* species combat fungal pathogens. These species not only enhance plant physiological defenses but also stimulate structural immunity, thereby providing robust protection against a wide spectrum of pathogenic microorganisms (Hashem et al., 2023). The efficacy of diverse *Trichoderma* strains against pathogenic microbes encountered in chili farming was demonstrated in Table 2. Yadav et al. (2023) observed that the treatment of seeds with *Trichoderma asperellum* and *T. harzianum* led to heightened cell wall robustness and the activation of defense-related genes within chili plants, resulting in effective mitigation of *Colletotrichum truncatum* infections. In field trials, Kim et al. (2023) demonstrated the efficacy of *Trichoderma atroviride* ATR697 and *T. longibrachiatum* LON701 in controlling anthracnose in chili plants, with LON701 exhibiting notably superior performance.

**Table 2:** *Trichoderma* against pests and pathogens in Chilies farming.

***Trichoderma***	**Pest/Pathogen**	**Remarks**	**Reference**
*Trichoderma asperellum and T. harzianum*	*Colletotrichum* *truncatum*	Treating seeds with *T. asperellum*, *T. harzianum*, or both enhanced cell wall strength via lignification and activated six defense-related genes—CaPDF1.2, SOD, APx, GPx, PR-2, and PR-5—in chili plants, effectively combating *C. truncatum* infections.	Yadav et al. (2023)
*Trichoderma atroviride* ATR697 and *T. longibrachiatum* LON701	*Colletotrichum acutatum*	Both ATR697 and LON701 strains effectively controlled anthracnose in chili plants. Notably, using LON701 resulted in a disease rate of 14 %, showing better performance compared to ATR697 strain as well as chemical and untreated controls.	Kim et al. (2023)
*Trichoderma harzianum* IPL/VT/102	*Fusarium oxysporum*	Field trials showed that applying strain IPL/VT/102 effectively managed Fusarium wilt in chili plants.	Sharma et al. (2023)
*Trichoderma hamatum* MHT1134	*Fusarium oxysporum*	Field tests demonstrated that strain MHT1134 can suppressed pepper Fusarium wilt, showing control comparable to the chemical hymexazol while significantly enhancing pepper yield.	Mao et al. (2020)
*Trichoderma harzianum*	*Fusarium oxysporum*	*Trichoderma harzianum* exhibits mycoparasitic activity by producing antibiotics, competing for nutrients and space, and penetrating and lysing the mycelium of *Fusarium oxysporum*.	Abdelaziz et al. (2022)
*Trichoderma longibrachiatum*	*Meloidogyne incognita and* *F. oxysporum*	Zinc nanoparticles synthesized by *Trichoderma longibrachiatum* alter defense-related gene expression in chili plants and deform *Fusarium oxysporum* hyphae, causing them to lose smoothness, swell, and crumble.	Ghareeb et al. (2024)
*Trichoderma asperelloides and* *T. asperellum*	*Colletotrichum gloeosporioides*	*Trichoderma asperelloides* SKRU-01 and *T. asperellum* NST-009 significantly inhibited the hyphal development of *Colletotrichum gloeosporioides* PSU-03	Boukaew et al. (2024)
*Trichoderma harzianum*	*Phytophthora capsici*	*T. harzianum* showed the highest antagonistic activity (42.86 % PICR) *in vitro* and was classified as class I on the Bell scale. Thus, along with three other *Trichoderma* spp., the species effectively controls the root necrosis caused by *P. capsici* in Manzano chili cultivation.	de Ita et al. (2021)
*Trichoderma virens* HZA14	*Phytophthora capsici*	Strain HZA14 likely employs various mechanisms to collaboratively hinder pathogens, with its production of gliotoxin potentially serving as a crucial metabolite in pathogen inhibition and the management of chili pepper blight, caused by *Phytophthora capsici*.	Tomah et al. (2020)
*Trichoderma albolutescens*	Pepper mottle virus	The purification of trichoderminol and trichodermin from *Trichoderma albolutescens* showed its can effectively prevent Pepper mottle virus infections in plants like tobacco and Chilies by a PepMoV-GFP based systemic host method.	Ryu et al. (2017)

Similarly, Sharma et al. (2023) documented the successful management of Fusarium wilt in chili plants through the application of *Trichoderma harzianum* IPL/VT/102, resulting in enhanced plant growth and yield. Mao et al. (2020) illustrated the suppressive effects of *Trichoderma hamatum* MHT1134 on pepper Fusarium wilt, achieving pepper yield enhancements comparable to chemical treatments. Abdelaziz et al. (2022) indicate that the combined effect of *T. harzianum* and *P. expansum* increased protection against fusarial wilt by 76.74 %. In comparison, *T. harzianum* alone provided 50 % protection, and *P. expansum* alone provided 17.64 % protection. The study suggests that several mechanisms contribute to the susceptibility of *F. oxysporum* to *T. harzianum* treatment. These mechanisms include the mycoparasitic activity of *T. harzianum*, its production of antibiotics, competition for nutrients and space, and its ability to penetrate and lyse the fusarial mycelium. Moreover, Ghareeb et al. (2024) stated that the zinc nanoparticles synthesized by *T. longibrachiatum* exhibit both anti-nematode and antifungal properties, effectively protecting plants against *Meloidogyne incognita* and *F. oxysporum*, particularly in chili plants. These nanoparticles function by altering the expression of defense-related genes in the chili plant. Additionally, it causes deformation of *F. oxysporum* hyphae, leading to a loss of smoothness, swelling, and crumbling of the hyphal structure.

*Trichoderma asperelloides* SKRU-01 and *T. asperellum* NST-009 exhibited remarkable and rapid growth, significantly impacting the hyphal development of *Colletotrichum gloeosporioides* PSU-03 through dual culture assay. This observation suggests a robust competition for space and nutrients, resulting in the inhibition and/or parasitization of the pathogen, thereby underscoring their potent antifungal properties. Furthermore, an in-planta mechanism revealed that these strains produce volatile compounds with activity against *C. gloeosporioides*, highlighting their potential for biocontrol applications in chili crops comparable to five chemical fungicides (Boukaew et al., 2024). Additionally, de Ita et al. (2021) underscored the effectiveness of *Trichoderma harzianum* in controlling root necrosis induced by *Phytophthora capsici* in Manzano chili cultivation. Tomah et al. (2020) proposed that *Trichoderma virens* HZA14 inhibits pathogens such as *Phytophthora capsici* through diverse mechanisms, potentially involving gliotoxin production. Lastly, Ryu et al. (2017) validated the capacity of *Trichoderma albolutescens* to efficiently prevent Pepper mottle virus infections in tobacco and chili plants utilizing a PepMoV-GFP based systemic host method.

Although *Trichoderma* spp. has primarily demonstrated its effectiveness against pathogenic microbes in chili farming, recent studies have indicated its potential for pest control in other crops. Islam et al. (2021) demonstrated that *Trichoderma harzianum* TMS623 exhibited high efficacy against aphids, both nymphs and adults, known to cause diseases in sugarcane woolly aphids. Similarly, *T. harzianum* has been shown to outperform *B. bassiana*, an entomopathogenic fungi, in eliminating aphids, suggesting its potential as a substitute for hazardous chemical pesticides such as malathion, thereby offering a more environmentally friendly approach to aphid biocontrol (Mukherjee and Ghosh, 2023). Additionally, Aldaghi et al. (2021) proposed that inoculating tomatoes with the *T. harzianum* Tr6 strain could induce resistance to whiteflies, resulting in reduced host preference, egg production, and honeydew secretion. These findings underscore the potential utilization of *Trichoderma* for pest management in chili farming, highlighting its multifaceted benefits beyond pathogen control.

**Fig. 5:**
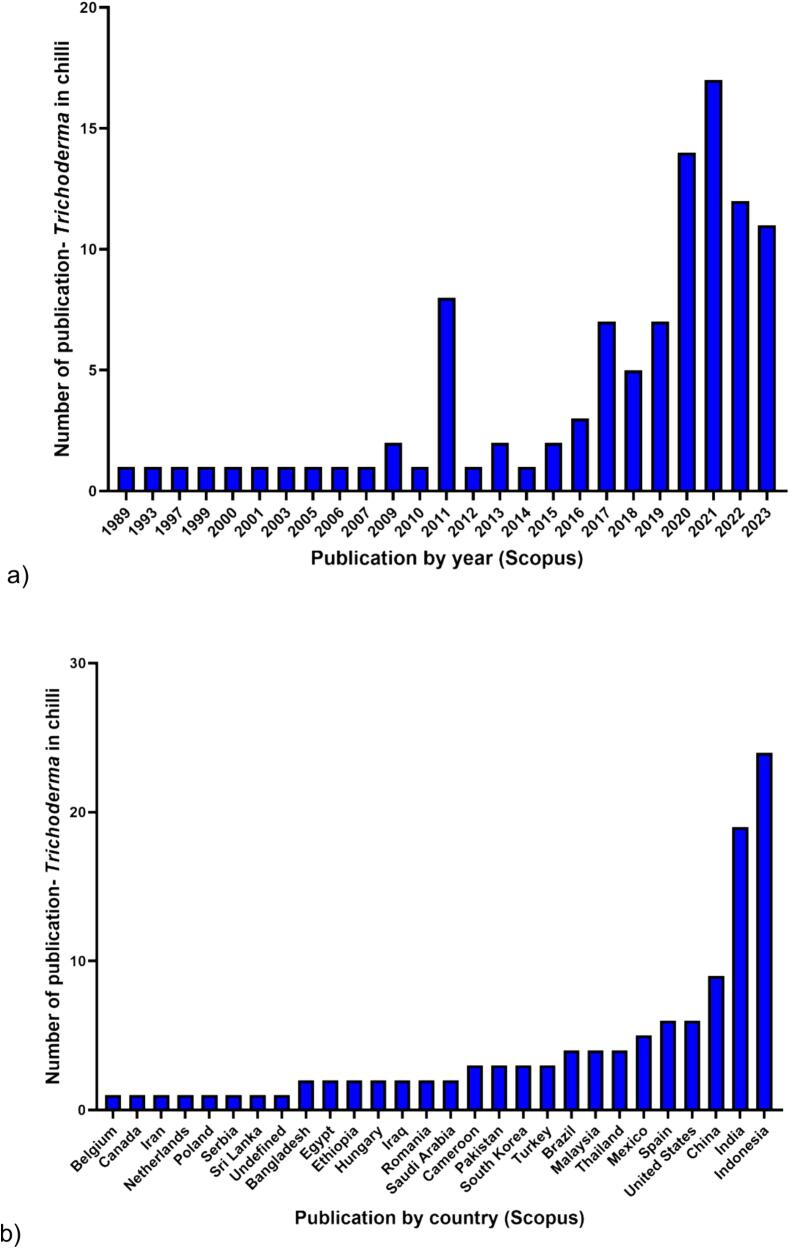
Data analytics on the application of *Trichoderma* spp. in chili production. These data were collected based on the results from the Scopus website (https://www.scopus.com) on February 26th, 2024. The search query “*Trichoderma* AND (Chili OR chili OR pepper)” was used to conduct an “article title” search on the Scopus website. The analysis focused on determining the number of publications by a) year and b) country.

Scopus-based data analytics on *Trichoderma* spp. in relation to chili production was showed in Fig. 5. These fungal genera were first discovered in chili in 1989, and since then, a total of 99 documents have been published. The highest number of publications on this topic was recorded in 2021, with 17 documents. Despite the increasing number of publications each year, research on *Trichoderma* in chili production remains relatively limited, suggesting that these microbes are not fully explored in the context of chili production. The Scopus database has identified 28 countries in connection with *Trichoderma* research in chili production. Indonesia has reported the highest number of publications related to *Trichoderma* in chili production, with 24 documents, followed by India and China with 19 and 8 documents, respectively. Malaysia had only 4 documents on this topic, which were published by Chin et al., 2022, Ong et al., 2015, Tangga et al., 2022, and Kwan and Wilson (2022).

### 4.3 Entomopathogenic fungi

Entomopathogenic fungi are parasitic microorganisms that can infect and kill insects/arthropods (Pedrini, 2022). *Metarhizium* and *Beauveria* species, often found in soil, manage natural arthropod populations, and develop intricate connections with plants. They are endophytes of plant roots, stems, and leaves (Jaber and Enkerli, 2017). These fungi kill the pest by attaching themselves to the insect's exoskeleton and secreting enzymes into its soft body parts to get nutrients for its growth (Sharma et al., 2020). Similar to *Trichoderma* spp., there is no record of the registration of entomopathogenic fungi as pesticides or insecticides from the Department of Agriculture Malaysia. However, the US Environmental Protection Agency (2003) and US Environmental Protection Agency (2006) indicated that *Metarhizium anisopliae* and *Beauveria bassiana* were considered insecticides and non-toxic to humans and mammals.

The efficacy of various entomopathogenic fungi against pests and pathogens in chili farming was demonstrated in Table 3. Francis and Manchegowda (2023) observed that *Beauveria bassiana* exhibited significant effectiveness against *Scirtothrips dorsalis* compared to *Metarhizium pingshaense* isolates, with the *B. bassiana* GKVK 01_06 isolate demonstrating notable virulence. In a related study, Mantzoukas et al. (2022) demonstrated the remarkable reduction in aphid populations achieved by *Metarhizium brunneum* at a concentration of 1 × 10^8^ conidia/mL. Furthermore, Soesanto et al. (2021) emphasized the role of secondary metabolites from entomopathogenic fungi in mitigating infections of *Fusarium oxysporum* f.sp. *capsici* in chili plants.

**Table 3:** Entomopathogenic fungi against pests and pathogens in Chilies farming.

***Trichoderma***	**Pest/Pathogen**	**Remarks**	**Reference**
*Metarhizium pingshaense* and *Beauveria bassiana*	*Scirtothrips dorsalis*	*B. bassiana* were highly effective against *S. dorsalis* compared to *M. pingshaense* isolates. The *B. bassiana* GKVK 01_06 isolate showed particularly strong virulence, with a low LC50 value of 2.8 × 10^4^ conidia/ml against *S. dorsalis*.	Francis and Manchegowda (2023)
*Beauveria bassiana* and *Metarhizium brunneum*	Aphids (*Myzus persicae*)	*M. brunneum* at a concentration of 1 × 108 conidia/mL resulted in a drastic reduction of aphid populations, nearly reaching zero levels.	Mantzoukas et al. (2022)
*Beauveria bassiana B10 and B16, Metarhizium anisopliae M16,* and *Lecanicillium lecanii L16*	*Fusarium oxysporum*	The secondary metabolites of entomopathogenic fungi reduced the infections of *Fusarium oxysporum* f. sp. capsici in chili plants at more than 48 %.	Soesanto et al. (2021)
*Beauveria bassiana*	Thrips (*Scirtothrips dorsalis*) and mites (*Polyphagotarsonemus latus*)	The LC50 values against *S. dorsalis* and *P. latus* revealed that the *B. bassiana* formulation exhibited the highest virulence, with the lowest LC50 values recorded at 1.58 × 10^5^ and 5.20 × 10^6^ spores mL^−1^, respectively.	Murugasridevi et al. (2021)
*Metarhizium anisopliae*	Viruses	The secondary metabolites from *M. anisopliae* demonstrated strong disease suppression by reducing the incubation period, viral disease intensity, and AUDPC by 34.22 %, 77.98 %, and 79.49 %, respectively.	Soesanto et al. (2020)
*Cordyceps fumosorosea*	Whitefly	*Cordyceps fumosorosea*, as an entomopathogenic fungi, holds promise as a viable alternative in combating invasive whiteflies, such as the pepper whitefly, in horticulture production within the United States.	Avery et al. (2020)
*Ophiocordyceps sobolifera* Cod-NB1302	*Colletotrichum capsici* and *C. gloeosporioides*	Two bioactive constituents, adenosine and cordytropolone, from the mycelial extract of *Ophiocordyceps sobolifera* Cod-NB1302, inhibited growth of both *Colletotrichum capsici* and *C. gloeosporioides.*	Jaihan et al. (2018)

Moreover, Murugasridevi et al. (2021) elucidated the significant virulence of *Beauveria bassiana* against thrips and mites, supported by the attainment of the lowest LC50 values. Similarly, Soesanto et al. (2020) showcased the efficacy of secondary metabolites from *Metarhizium anisopliae* in effectively suppressing viral diseases in chili plants. Additionally, Avery et al. (2020) stated *Cordyceps fumosorosea* as a promising alternative for combating invasive whiteflies in U.S. horticulture production. Finally, Jaihan et al. (2018) made a noteworthy discovery regarding *Ophiocordyceps sobolifera* Cod-NB1302, which exhibited inhibitory effects on the growth of *Colletotrichum capsici* and *C. gloeosporioides* in chilies plantation through bioactive constituents derived from its mycelial extract.

Table 3 indicates that research on entomopathogenic fungi for pest control in chili plants is limited, despite the need to manage new and emerging pests. Currently, large amounts of chemical pesticides are used, which is unsustainable and harmful to the environment and human health. Entomopathogenic fungi show great potential for future pest control in chili cultivation. They offer a more sustainable and eco-friendlier alternative, reduce chemical residues in food, and help preserve beneficial insect populations. Increasing research and development in this area could lead to more effective and widely adopted biological control methods in agriculture.

**Fig. 6:**
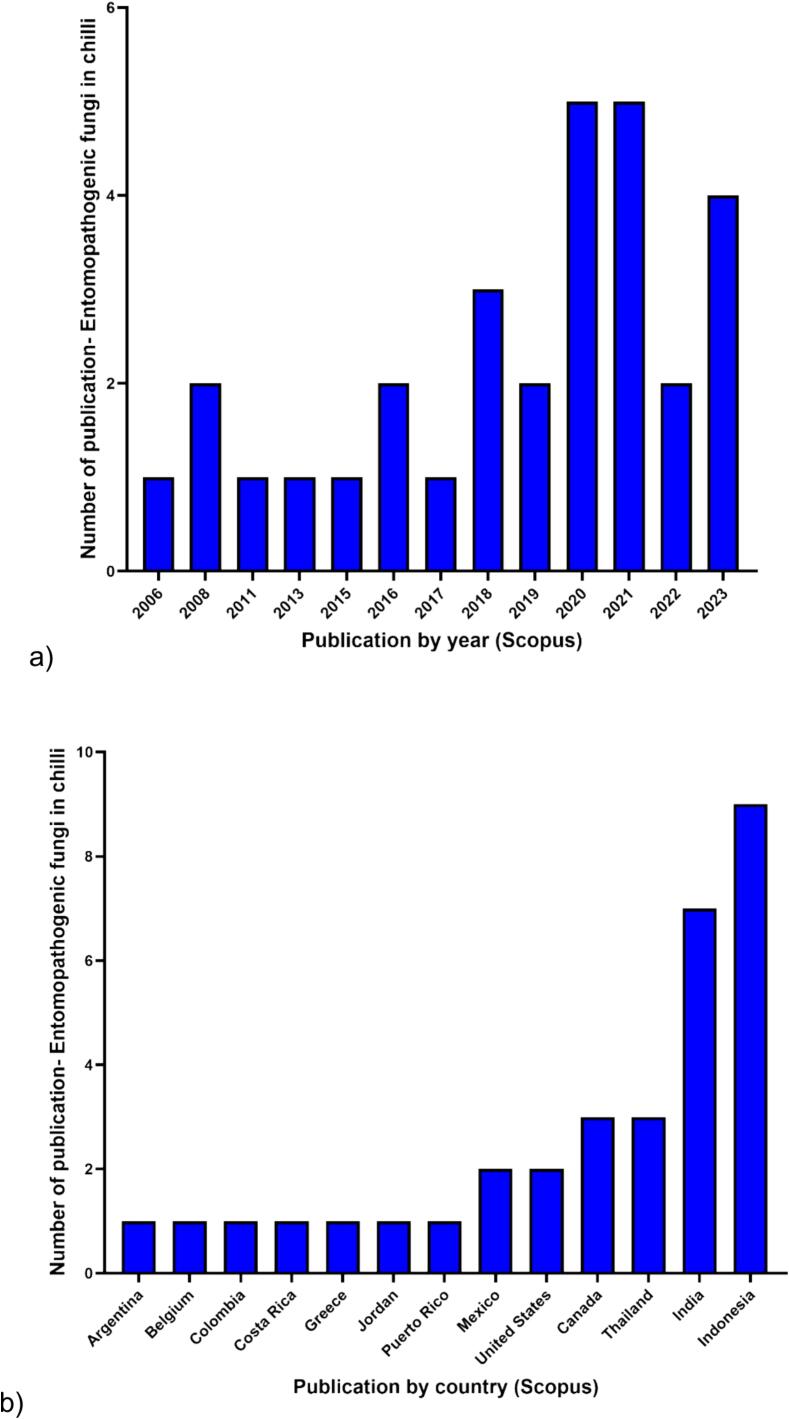
Data analytics on applying entomopathogenic fungi in Chili production. The number of publications was collected based on the result from the Scopus website (https://www.scopus.com) on February 26th, 2024. “((Entomopathogenic AND fungi) OR (Entomopathogenic AND fungus) OR *Metarhizium* OR *Beauveria*) AND (Chili OR chili OR pepper)” was used as search query in Scopus website.

Moreover, Scopus-based data analytics on entomopathogenic fungi in relation to chili production was showed in Fig. 6. Two genera, *Metarhizium* and *Beauveria*, were included in the query search due to their commercial recognition as entomopathogenic fungi. Reports of these fungi in chili cultivation began in 2006, with a total of 29 documents published since then. However, the highest number of reported documents on these entomopathogenic fungi in chili production was in 2020 and 2021, with only 5 documents for each year, respectively. Additionally, based on publications by country, only twelve countries have published research on entomopathogenic fungi in relation to chili in the Scopus database. Indonesia showed the highest number of publications at 9, followed by India and Thailand with 5 documents each. Moreover, there have been no publications reported by Malaysia on entomopathogenic fungi in chili production. This evaluation demonstrates that the use of entomopathogenic fungi in chili cultivation has received limited attention. However, more research into this strategy is highly encouraged, as these fungi have the potential to effectively control pests. If entomopathogenic fungi can eliminate insects in chili farming, infections caused by viruses can also be significantly reduced.

## 5 Conclusion

In conclusion, microbe-based biocontrol, including *Bacillus* spp., *Trichoderma* spp., and entomopathogenic fungi, presents viable alternatives to chemical pesticides for managing pests and pathogens in chili plantations. These alternatives offer the potential for safe and non-toxic treatments for growers and chili crops alike. Furthermore, an analysis of research output on *Bacillus* spp., *Trichoderma* spp., and entomopathogenic fungi in chili cultivation, conducted using the Scopus database, revealed fewer than 30 publications per year. Notably, India, as the largest chili producer globally, has emerged as one of the top three countries publishing research on all three microbial agents related to chili. However, despite ongoing research efforts, the data indicates that investigations into the efficacy of microbes as biocontrol agents in chili farming are still in progress. Therefore, it is imperative to disseminate this concept widely, particularly among farmers, to diminish reliance on chemical pesticides across global chili farms. This shift towards biocontrol methods promises not only enhanced sustainability but also reduced environmental and health risks associated with conventional pesticide use.

**Funding**

This research received no specific grant from public, commercial, or not-for-profit funding agencies.

## CRediT authorship contribution statement

**Muhamad Firdaus Syahmi Sam-on:** Writing – original draft, Project administration, Methodology, Investigation, Formal analysis, Data curation, Conceptualization. **Shuhaimi Mustafa:** Writing – review & editing, Visualization, Supervision. **Mohd Termizi Yusof:** Writing – review & editing, Visualization, Supervision. **Amalia Mohd Hashim:** Writing – review & editing, Visualization, Supervision. **Ku Nur Azwa Ku Aizuddin:** Writing – review & editing, Visualization.

## Declaration of competing interest

The authors declare that they have no known competing financial interests or personal relationships that could have appeared to influence the work reported in this paper.
